# Supplementary material for: Evolution of sexual conflict in scorpionflies
Source: eLife. 2022 Feb 11;11:e70508. doi: 10.7554/eLife.70508 (PMC8983043; doi:10.7554/eLife.70508)
Supplement: Supplementary file 1. — T5, T6, tergites 5, 6. Measurements are approximate, based on scale bars in published figures as specified. [file elife-70508-supp1.docx]

| *Species name* | NO-PO | C-R1 | Relative length of NO | | Reference: Fig. |  |
| --- | --- | --- | --- | --- | --- | --- |
| *Neopanorpa lui* | 0.4 | 0.4 | shorter than PO | Tong and Hua 2019: Fig. 4a; wing length: Wang and Hua 2018c | | |
| *Neopanorpa carpenteri* | 0.4 | 0.4 | to PO | Tong and Hua 2019: Fig. 4b; wing length: Wang and Hua 2018c | | |
| *Leptopanorpa charpentieri* | 0.35 | 0.31 | to PO | Wang and Hua 2020: Fig. 6c, n | | |
| *Leptopanorpa nematogaster* | 0.25 | 0.25 | to PO | Wang and Hua 2020: Fig. 7c, n | | |
| *Leptopanorpa majapahita* | 0.30 | 0.31 | just beyond PO | Wang and Hua 2020: Fig. 5b, k | | |
| *Neopanorpa longiprocessa* | 0.35 | 0.4 | to end of T5 | Tong and Hua 2019: Fig. 4c; wing length: Wang and Hua 2018c | | |
| *Neopanorpa setigera* | 0.4 | 0.4 | to end of T6 | Wang and Hua, 2018b: Fig 1B, F | | |
