# Supplementary material for: Evolution of sexual conflict in scorpionflies
Source: eLife. 2022 Feb 11;11:e70508. doi: 10.7554/eLife.70508 (PMC8983043; doi:10.7554/eLife.70508)
Supplement: Supplementary file 2. — AH, anal horn; NO, notal organ; PA, postnotal area; PO, postnotal organ; A5, A6, abdominal segments 5, 6. [file elife-70508-supp2.docx]

|  |  |  |  |
| --- | --- | --- | --- |
| Species | **Remarks on abdomen** | **Age, Locality** | **References** |
| HOLCORPIDAE | | | |
| *Conicholcorpa longa* Zhang, Shih and Ren, 2021 | NO+PO absent; A6-A8 greatly elongate [swellings on hind tarsomeres] | Middle Jurassic, Yanliao Biota, North Eastern China | Zhang et al., 2021 |
| *Conicholcorpa stigmosa* Li, Shih, Wang and Ren, 2017 | NO +PO absent or very small (abdomen covered with wings) | latest Middle Jurassic Daohugou, Inner Mongolia, China | Li et al., 2017 |
| *Holcorpa dillhoffi* Archibald, 2010 | paired anal spurs on A6, dorsally (A1-A5 poorly pres., NO+PO invisible, probably absent) | Ypresian (Early Eocene)  McAbee beds of British Columbia | Archibald, 2010 |
| *Holcorpa maculosa* Scudder, 1878 | paired anal spurs on A6, dorsally (A1-A5 poorly pres., NO+PO invisible, probably absent) | Late Eocene of Florissant, Colorado, USA | Carpenter, 1931; Archibald, 2010 |
| INCERTAE SEDIS (close to Holcorpidae) | | | |
| *Miriholcorpa forcipata* Wang, Shih and Ren, 2013 | abdomen without modifications | late Middle Jurassic Daohugou, Inner Mongolia, China | Wang et al., 2013 |
| *Fortiholcorpa paradoxa* Wang, Shih and Ren, 2013 | NO+PO absent or very small (abdomen covered with wings) | late Middle Jurassic, Daohugou, Inner Mongolia, China | Wang et al., 2013 |
| ORTHOPHLEBIIDAE | | | |
| *Orthophlebia extensa* Martynov, 1937 | NO+PO absent or very small (abdomen covered with wings) | Middle Jurassic, Yanliao Biota | Zhang et al., 2021 |
| *Gigaphlebia riccardii* (Petrulevičius and Ren, 2012) | NO+PO absent | Middle Jurassic | Petrulevicius and Ren, 2012; Soszyńska-Maj et al., 2018 |
| *Orthophlebia nervulosa* Qiao, Shih and Ren, 2012 | NO+PO absent | Jiulongshan Formation,  Middle Jurassic; Daohugou Village,  Inner Mongolia, China. | Qiao et al., 2012 |
| *Orthophlebia elenae* Willmann and Novokshonov 1998 | abdomen missing | Upper Jurassic, Karatau  Middle Jurassic, Yanliao Biota | Willmann and Novokschonov, 1998;  Zhang et al., 2021 |
| *Orthophlebia heidemarie* Willmann and Novokshonov 1998 | NO & AH, see this paper Fig. 5 | Upper Jurassic, Karatau | Willmann and Novokschonov, 1998 |
| *Orthophlebia longicauda* Willmann and Novokshonov 1998 | NO+PO absent | Upper Jurassic, Karatau | Willmann and Novokshonov, 1998 |
| *Orthophlebia chinensis* Soszyńska-Maj, Kopeć and Ren, 2020 | NO+PO absent or very small | Middle-Late Jurassic, Daohugou village,  Inner Mongolia, China | Soszyńska-Maj  et al., 2020 |
| *Juraphlebia eugeniae* Soszyńska-Maj and Krzemiński, 2018 | NO+PO absent | Middle-Late Jurassic, Daohugou village,  Inner Mongolia, China | Soszyńska-Maj,  et al., 2020 |
| *Burmorthophlebia multiprocessa* gen. et sp. nov. | Small NO, PA1, PA2, AH | Kachin amber, Myanmar; earliest Cenomanian (98.8 ± 0.62 Ma), Late Cretaceous | This paper; Figs 2, 5 |
| Protorthophlebiidae | | | |
| *Protorthophlebia punctata* Soszyńska-Maj,  Krzemiński and Kopeć, 2020 | NO+PO absent | Middle-Late Jurassic, Daohugou village,  Inner Mongolia, China | Soszyńska-Maj  et al., 2020 |
| Panorpidae | | | |
| *Baltipanorpa damzeni* Krzemiński and Soszyńska-Maj, 2012 | Very long NO+PO | Baltic amber, middle Eocene (ca. 45 Ma). | Krzemiński and Soszyńska-Maj, 2012; this paper, Fig 6 |
| *Baltipanorpa oppressiva* sp. nov. | Very long NO+PO | Baltic amber, middle Eocene (ca. 45 Ma). | This paper; Figs 1, 6 |
| Cantabridae fam. nov. |  |  |  |
| *Cantabra soplao* gen. et sp. nov. | Small PO, possibly very small NO (although not visible) | El Soplao amber outcrop; middle Albian, Early Cretaceous | This paper; Figs 3,4 |

**References not cited in the main text:**

Martynov, A. 1937. Liassic insects from Shurab and Kisyl-Kiya. Part 1. Various orders except Blattodea and Coleoptera. *Trudy Paleontologicheskogo Instituta Akademii Nauk SSSR* **7**: 1*–*232.

Petrulevičius J, Ren D. 2012. A new species of "orthophlebiidae" (Insecta: Mecoptera) from the Middle Jurassic of Inner Mongolia, China. *Revue de Paléobiologie* **31**: 311*–*315.
